# GIGANTEA mediates WRKY-dependent transcriptional activation of leaf senescence

**DOI:** 10.1080/15592324.2026.2639486

**Published:** 2026-03-02

**Authors:** Bongsoo Choi, Gyeongik Ahn, Kyujin Kim, Jin Sung Huh, Jung Jun Ma, Woe-Yeon Kim

**Affiliations:** a Division of Applied Life Science (BK21 Four), PBRRC, PMBBRC, RILS & IALS, Gyeongsang National University, Jinju, Republic of Korea

**Keywords:** GIGANTEA, leaf senescence, WRKY transcription factor, *ORE1*, *SAG12*

## Abstract

Leaf senescence is a genetically programmed process that integrates developmental and environmental cues to optimize nutrient remobilization. GIGANTEA (GI), a circadian regulator with diverse functions in flowering, temperature, and stress response pathways, has also emerged as a positive regulator of age-dependent leaf senescence. However, the molecular link between the GI and senescence-associated transcription factors is still poorly defined. Here, we investigated the interactions between GI and WRKY transcription factors from the WRKY family, which have been previously reported as positive regulators of senescence in *Arabidopsis*. GI physically interacts with WRKY45 and WRKY75 transcription factors in a yeast-II-hybrid assay. We further analyzed the interaction between WRKY75 and GI *in vivo* using co-immunoprecipitation (Co-IP) assay in a transient expression system in *Nicotiana benthamiana*. Genetic analyses using *Arabidopsis* mutant lines further revealed that GI is required for the WRKY75-mediated promotion of age-dependent leaf senescence. Consistent with these phenotypes, WRKY75 positively regulates *SAG12* expression during senescence in a GI-dependent manner. Together, our findings position GI as an integrative component of WRKY-driven senescence pathways, expanding its functional role in coordinating age-dependent leaf senescence.

## Background

Leaf senescence is not a passive outcome of aging but an actively regulated, genetically programmed process shaped by both intrinsic developmental cues and external environmental signals. It involves the controlled breakdown of cells, tissues, and organs, most prominently in leaves, the main organs for photosynthesis and biomass accumulation.^1-3^ In annual plants, nutrient remobilization during leaf senescence is crucial for supporting seed development and maximizing reproductive success. In perennial species, these nutrients are redirected to storage organs such as roots or stems, supporting regrowth in subsequent seasons.^2^ Thus, the precise timing of senescence onset is essential for plant fitness, as it is finely tuned to developmental programs with environmental conditions.^4^

Plant-specific transcription factors such as NAC (NAM, ATAF, and CUC) and WRKY regulate downstream gene expression, controlling plant leaf senescence.^5^ NAC family of transcription factors are intensively studied based on its importance regulating senescence.^6-12^ Especially, ORESARA1 (ORE1, also named NAC2, NAC6, and NAC092) is regarded as a regulatory hub of senescence.^8^ ORE1 induces a suite of *senescence-associated genes* (*SAGs*) to activate downstream senescence programs. The senescence-induced nucleic acid-degrading enzyme BIFUNCTIONAL NUCLEASE1 (BFN1) is activated by ORE1 through its binding to the *BFN1* promoter.^13^ Although the binding of ORE1 to the *SAG12* promoter has not been confirmed,^8^ the senescence-induced protease *SAG12* is also upregulated in an ORE1-dependent manner. WRKY transcription factors constitute another major regulatory module that integrates aging, hormonal, and environmental signals to modulate senescence. Although *Arabidopsis* encodes 72 WRKY transcription factors with substantial functional redundancy, several WRKYs have been characterized as positive regulators of age-dependent senescence.^14^ For example, WRKY1 integrates flowering pathways, nitrogen deficiency responses, and salicylic acid (SA) signaling, and WRKY45 is linked to the gibberellin (GA) signaling pathway to promote age-dependent leaf senescence.^15^^,^^16^ WRKY53 is a positive regulator whose expression rises rapidly at the onset of early senescence.^17^ WRKY75 promotes senescence by coordinating SA signaling with H₂O₂-mediated oxidative pathways and directly binding to the promoter of *SAG12* through the WRKY binding motif.^18^ The expression of these *WRKYs* was induced by leaf age.^15-20^

The circadian clock functions as a central coordinator that harmonizes internal metabolic status with external environmental cues and also orchestrates the temporal regulation of leaf senescence.^4^ The morning-phased core clock component CIRCADIAN CLOCK ASSOCIATED 1 (CCA1) negatively regulates leaf senescence by directly repressing *ORESARA1* (*ORE1*) while activating *GOLDEN2-LIKE TRANSCRIPTION FACTOR 2* (*GLK2*) through direct promoter binding.^21^ PSEUDO-RESPONSE REGULATOR 9 (PRR9) positively regulates leaf senescence by activating *ORE1* expression.^12^ The evening-phased clock component GI enhances senescence progression by localizing to the nucleus and associating with the *ORE1* promoter to induce gene expression, whereas EARLY FLOWERING 4 (ELF4) inhibits this activity by sequestering the GI within subnuclear compartments.^22^

GI is increasingly recognized as a multifunctional molecular scaffold that coordinates diverse biological pathways with the circadian clock through direct interaction with transcription factors.^23-27^ For example, GI interacts with the flowering repressors SHORT VEGETATIVE PHASE (SVP) and TEMPRANILLO1/2 (TEM1 and TEM2) to facilitate FT promoter activation in photoperiodic flowering regulation.^28^ In addition, GI associates with CYCLING DOF FACTOR 1 (CDF1) to enhance the FKF1-dependent degradation of CDF1, thereby promoting *CONSTANS* (*CO*) expression.^29^ Although no canonical protein domain has been identified in the GI and lacks any known DNA-binding domain, the GI plays a role in senescence promoting *ORE1* expression associated with its promoter.^22^ However, the interconnection between the GI and senescence-related transcription factors is still poorly understood. WRKY transcription factors are well-established regulators of senescence.^9^ In this study, we uncover a previously uncharacterized regulatory connection between GI and WRKY transcription factors in age-dependent leaf senescence. By elucidating how GI integrates with WRKY-mediated transcriptional programs in senescence, our work provides important mechanistic insights into how plants coordinate developmental aging with environmental rhythms. These findings advance the understanding of senescence as a dynamic and clock-synchronized process and highlight a new molecular axis that could be used to optimize crop productivity, stress resilience, and seasonal adaptation.

## Results

### GI interacts with senescence-associated WRKY transcription factors and is essential for WRKY75-mediated leaf senescence

**Figure 1.**
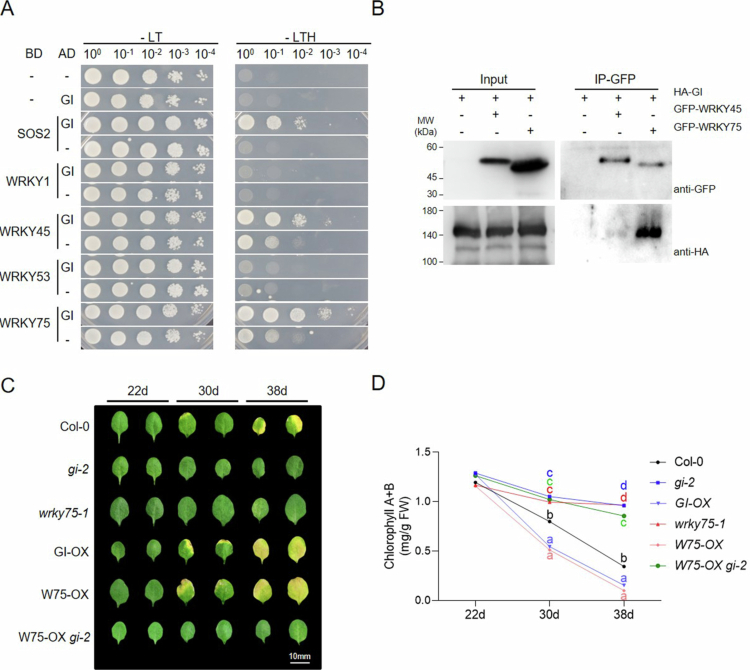
GI interacts with senescence-regulating WRKY transcription factors and is necessary for WRKY75-dependent leaf senescence. (A) Yeast two-hybrid assay for the interaction between GI and four WRKY transcription factors (WRKY1, 45, 53, and 75). (B) Co-immunoprecipitation assays in *N. benthamiana* leaves transiently expressing *35Spro:HA-GI* (*HA-GI*) and/or *35Spro:GFP-WRKY45* (*GFP-WRKY45*), *35Spro:GFP-WRKY75* (*GFP-WRKY75*). Total protein extracts (Input; left panels) and proteins immunoprecipitated with an anti-GFP antibody (IP: GFP; right panels) were subjected to immunoblot analysis. In both Input and IP samples, the upper panels show the detection of GFP-tagged proteins, whereas the lower panels show the detection of HA-tagged proteins using an anti-HA antibody. (C) Representative phenotypes of Col-0 (wild-type), *gi-2*, *wrky75-1*, *GI-OX*, *WRKY75-OX*, and *WRKY75-OX gi-2* plants at 22, 30, and 38 d after germination (DAG). For each DAG, the third and fourth rosette leaves were placed for comparison. (D) Quantification of total chlorophyll content (mg/g FW) in the third and fourth leaves from the indicated genotypes. The values are means ± SD of three independent biological replicates. Significance was determined using one-way ANOVA followed by Tukey's HSD post hoc (*p* < 0.033) for the genotypes at each DAG (30 d and 38 d).

To investigate the connection between GI and WRKY transcription factors in senescence, we first examined the direct interaction of GI with four known senescence-associated WRKY transcription factors: WRKY1, WRKY45, WRKY53, and WRKY75 using yeast two-hybrid assay. The full-length *GI* coding region was cloned into prey vector tagging with activation domain (AD), and *SOS2*, *WRKY1, 45, 53*, and *75* were inserted into bait vector tagging with DNA-binding domain (BD). Among the tested candidates, yeast co-transformed with GI and WRKY45 or WRKY75 exhibited enhanced growth on TDO (–Leu/–Trp/–His) selective media compared with the prey-alone controls, indicating that GI interacts with both WRKY45 and WRKY75 in the yeast II hybrid system. Although WRKY45 and WRKY75 displayed self-activation activity, GI-dependent enhancement was still evident (Figure 1A). *SOS2* was used as a positive control for interaction.^30^ To further confirm the interaction *in planta*, we performed co-immunoprecipitation (Co-IP) assays in *Nicotiana benthamiana* leaves transiently co-expressing *HA-tagged GI* (*HA-GI*) and *GFP-tagged WRKY45* (*GFP-WRKY45*) or *WRKY75* (*GFP-WRKY75*). GFP-WRKY45 and GFP-WRKY75 were immunoprecipitated using an anti-GFP antibody. HA-GI was co-immunoprecipitated with GFP-WRKY75, indicating that GI makes a complex with WRKY75, but not with WRKY45, *in planta* (Figure 1B).

To examine whether this molecular association influences leaf senescence, we evaluated age-dependent leaf senescence phenotypes in Col-0 (WT) with *gi-2, wrky75-1*, *GI-OX*, *WRKY-OX* single mutants, and *WRKY-OX gi-2* double mutants at three developmental stages: 22, 30, and 38 d after germination (DAG). The third and fourth rosette leaves from each genotype were examined to assess the progression of age-dependent senescence (Figure 1C). As reported, both *gi-2* and *wrky75-1* single mutants exhibited a pronounced stay-green phenotype, with visibly delayed leaf yellowing compared to WT plants,^22^^,^^31^ confirming that both WRKY75 and GI positively regulate leaf senescence. Up to 38 d after germination, no visible leaf yellowing was observed in any of the single mutants, whereas WT plants gradually exhibited increased yellowing 30 DAG from the 3rd rosette leaf and clearly seen leaf yellowing of both the 3rd and 4th rosettes at 38 DAG. *GI-OX* and *WRKY-OX* lines showed more rapid progression of senescence compared to WT plants. Interestingly, the *WRKY75-OX gi-2* line showed a delayed senescence phenotype comparable to that of the *gi-2* mutant delayed up to 38 d. To quantify these observations, the total chlorophyll content was measured in the third and fourth leaves at each time point. Chlorophyll contents of the *WRKY75-OX gi-2* crossed line, staying from ~1.3 mg/g FW at 22 DAG to ~0.85 mg/g FW at 38 DAG. The *gi-2* and *wkry75-1* stayed from ~1.3 mg/g FW at 22 DAG to ~1.0 mg/g FW at 38 DAG, showing delayed chlorophyll reduction compared with WT (~1.2 mg/g FW at 22 DAG to ~0.32 mg/g FW). In contrast, the chlorophyll content in the *GI-OX* and *WRKY75-OX* lines significantly declined from 30 DAG (~0.56 mg/g FW) and decreased to 38 DAG (~0.17 mg/g FW) (Figure 1D). These results suggested that GI is required for WRKY75-mediated promotion of age-dependent leaf senescence in *Arabidopsis*.

### GI-WRKY75 regulatory module controls *SAG12* expression during senescence

**Figure 2.**
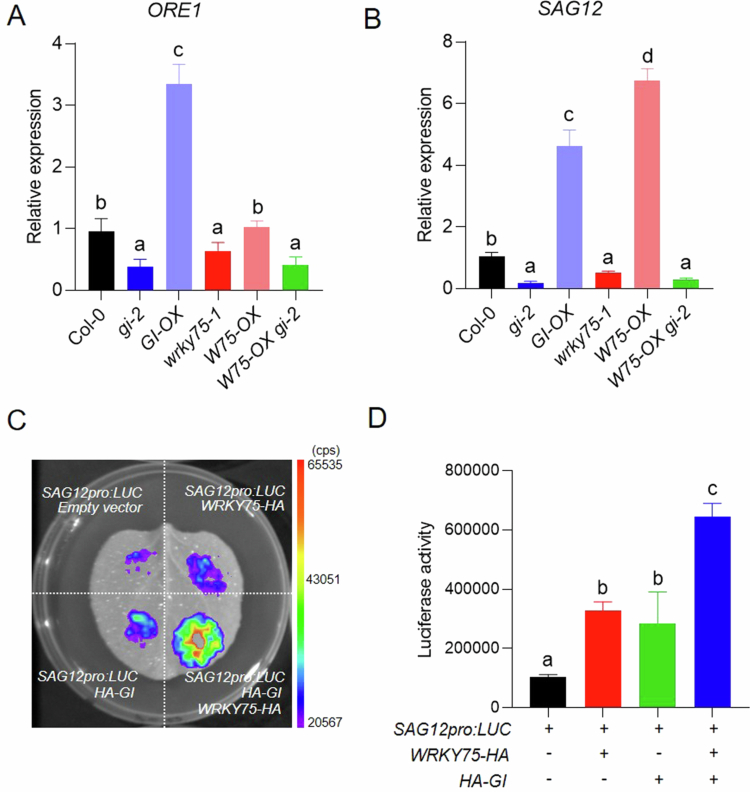
GI-WRKY75 regulatory module controls SAG12 expression during leaf senescence. (A and B) Relative transcript levels of *ORE1* (A) and *SAG12* (B) were measured by qRT-PCR in the third and fourth rosette leaves of Col-0, *gi-2*, *wrky75-1*, *GI-OX*, *WRKY75-OX*, and *WRKY75-OX gi-2* plants at 30 DAG. The relative expression was normalized by housekeeping gene *PP2A*. The data represent mean ± SD of three biological replicates. Significance was determined using one-way ANOVA followed by Tukey's HSD post hoc (*p* < 0.033) among the genotypes. (C and D) Regulation of *SAG12* promoter activity by GI and WRKY75 was measured by luciferase (LUC) reporter assay. (C) *N. benthamiana* leaves were divided into four sectors and infiltrated with equal OD₆₀₀ Agrobacterium mixtures containing: *SAG12pro:LUC* with *empty vector* (negative control), *SAG12pro:LUC* with *35Spro:WRKY75-HA*, *SAG12pro:LUC* with *35Spro:HA-GI*, or *SAG12pro:LUC* with *35Spro:HA-GI* and *35Spro:WRKY75-HA*. LUC activity image was captured using imaging system (NightSHADE LB 985 plant imaging system; Berthold Technologies). (D) Luciferase activity was quantified by Indigo software (v.2.0.5.0; Berthold Technologies). The data represent mean ± SD from four biological replicates. Statistical significance was assessed by one-way ANOVA with Tukey's HSD post hoc (*p* < 0.033).

To assess how GI and WRKY75 are interconnected in regulating gene expression of age-dependent senescence, we analyzed the transcript levels of *ORE1* and *SAG12* in WT, *gi-2*, *wrky75-1*, *GI-OX*, *WRKY75-OX*, and *WRKY75-OX gi-2* plants using quantitative real-time PCR (qRT-PCR). Total RNA was extracted from the third and fourth rosette leaves at 30 DAG, which is the transition time point of senescence progression in our growth condition. In a previous report, the GI protein promotes *ORE1* transcription as an upstream positive regulator.^22^ The *ORE1* transcript level was significantly downregulated in the *gi-2* mutant and was upregulated by approximately 3-fold in the *GI-OX* line. In contrast, *ORE1* expression showed no significant changes in either the *wrky75-1* mutant or the *WRKY75-OX* line, suggesting that *ORE1* expression may not be a direct target of the GI-WRKY75 module (Figure 2A). We next examined the expression level of *SAG12*, a well-known downstream target of WRKY75. WRKY75 binds to the *SAG12* promoter and activates the transcription of *SAG12.*^31^ Consistent with previous reports, the *wrky75-1* mutant displayed significantly reduced *SAG12* expression, whereas *SAG12* transcript levels increased by ~7-fold in the *WRKY75-OX* line compared to WT. Interestingly, the *gi-2* mutant also showed significant decreased *SAG12* expression, and the *GI-OX* line showed a ~5-fold increase compared to WT, indicating that both GI and WRKY75 contribute to *SAG12* activation. Moreover, *WRKY75-OX gi-2* double mutants showed a similar expression level with *gi-2,* suggesting that WRKY75 requires GI to fully activate *SAG12* expression (Figure 2B).

**Figure 3.**
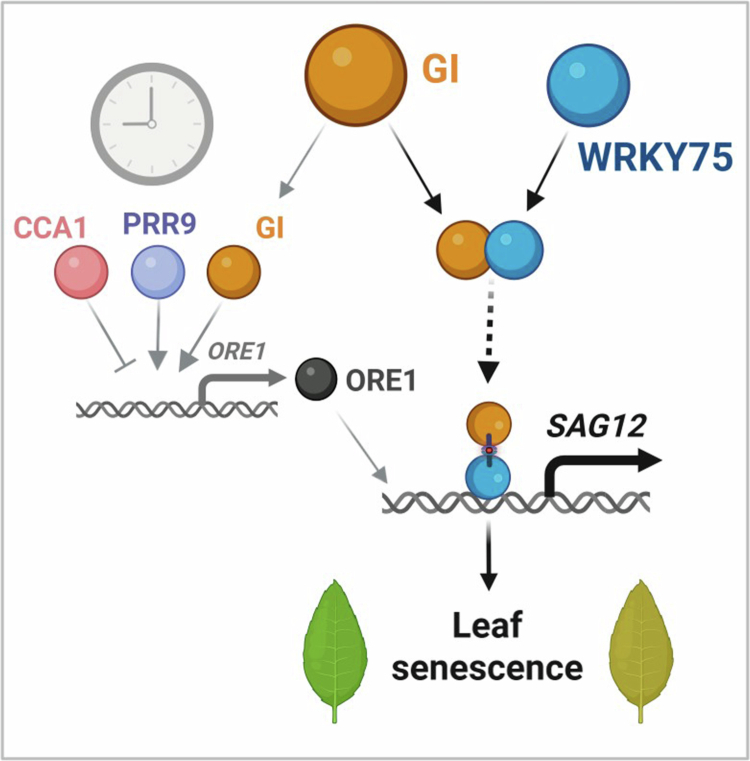
Working model linking the circadian clock to leaf senescence through the GI-WRKY75 module. GI promotes leaf senescence by bifurcated regulation of *SAG12* expression, acting through ORE1-dependent transcriptional activation and through direct interaction with and activation of WRKY75. The black lines indicate the molecular relationships identified in this study, whereas the gray lines represent previously reported regulatory relationships among circadian clock components, including CCA1,^21^ PRR9,^12^ and ELF4.^22^ The schematic illustration was generated using BioRender (https://biorender.com).

To further determine whether GI enhances WRKY75-mediated transcriptional activation of *SAG12*, we performed a transient luciferase assay using the *SAG12* promoter. A 1-kb region upstream of the *SAG12* transcription start site, containing multiple predicted W-box motifs (T/CTGACC/T), was cloned upstream of a firefly luciferase (LUC) reporter. The reporter construct was co-infiltrated into *Nicotiana benthamiana* leaves with effector plasmids encoding *WRKY75*, *GI*, or both, and LUC activity was measured 48 h after agro-infiltration (Figure 2C). WRKY75 expression alone significantly increased LUC activity driven by *SAG12* promoters compared to the empty vector control, confirming its role as a transcriptional activator. GI expression alone also increased LUC activity. Notably, co-expression of GI with WRKY75 led to a significant increase in LUC activity beyond that observed with WRKY75 or GI alone (Figure 2D). These results suggest that the co-expression of GI with WRKY75 synergistically enhances the transcriptional activation of *SAG12*. Based on our findings, a working model connecting circadian clock regulation to leaf senescence via the GI–WRKY75 module is proposed (Figure 3).

## Summary

Our findings establish GI as an essential regulatory hub that enables WRKY75-mediated activation of age-dependent leaf senescence. The physical interaction between GI and WRKY75 suggests a direct regulatory link between GI and WRKY75 (Figure 1A and B). In the *gi-2* mutant, *WRKY75* overexpression fails to promote senescence, demonstrating that WRKY75 can accelerate senescence in the presence of GI (Figure 1C and D). This data suggests that WRKY75-dependent senescence promotion through downstream gene expression is GI dependent. At the molecular level, GI specifically modulates *ORE1* expression, while both GI and WRKY75 are required for full activation of the downstream senescence marker *SAG12* (Figure 2A and B). Moreover, GI and WRKY75 form a complex *in vivo* and synergistically activated the *SAG12* promoter in transient assays, indicating that GI enhances WRKY75's transcriptional activity (Figure 2C and D). Collectively, these results define the GI as a core integrator that links circadian timing to WRKY-driven transcriptional activation during senescence (Figure 3). Interestingly, there is growing evidence for reciprocal regulation between the circadian clock and senescence. Senescence can provide feedback to alter clock periodicity, while clock components influence the expression of *SAGs*. Further work will advance our understanding of how circadian timing shapes senescence-regulatory networks and how GI and WRKY factors are incorporated into this temporal framework.

## Materials and methods

### Plant materials and growth conditions

All the mutant and transgenic *Arabidopsis thaliana* lines used in this study are in the Columbia (Col-0) background. The *wrky75-1* mutant (SALK_101367) is a loss-of-function T-DNA insertion line, with the insertion located in the first exon of the *WRKY75* gene.^33^*gi-2* is a loss-of-function allele caused by a point mutation that introduces a premature stop codon in the *GI* gene.^32^ The *35Spro:WRKY75 (WRKY75-OX)*^33^ and *35Spro:HA-GI* (*GI-OX*)^30^ overexpression lines are generated in the each *wrky75-1* and *gi-2* single mutant backgrounds, respectively. The *WRKY75-OX gi-2* double mutant was generated by genetic crossing. Seeds were surface sterilized with 30% (v/v) commercial bleach for 5 min, rinsed five times with sterile distilled water, and stratified for 3 d at 4 °C in darkness. Seedlings were germinated on Murashige and Skoog (MS) medium containing 1% (w/v) sucrose and 0.7% (w/v) agar under long-day (LD) conditions (16 h light/8 h dark, 23 °C, 100 µmol photons m⁻² s⁻¹ cool-white light, 60% relative humidity). Eight-day-old seedlings were transplanted to soil and grown under the same LD conditions until sampling at the indicated developmental stages.

### Leaf senescence assay and chlorophyll quantification

Age-dependent senescence was analyzed using the third and fourth rosette leaves collected at 22, 30, and 38 d after germination (DAG). Leaf yellowing was visually recorded. For chlorophyll quantification, chlorophyll was extracted using 95% ethanol (v/v) from plant leaves at 23 °C overnight shaking in the dark. The absorbances at 649 nm and 665 nm were measured using a spectrophotometer and calculated as described in a previous study.^34^ Statistical data represent mean ± SD of three biological replicates. Significance was determined using one-way ANOVA followed by Tukey's HSD post hoc (*p* < 0.033). Graphs were generated with GraphPad Prism 8.

### RNA extraction and quantitative RT-qPCR analysis

Total RNA was extracted from third and fourth rosette leaves at 22, 30, and 38 DAG using the Plant RNA mini-kit (QIAGEN). First-strand cDNA was synthesized from 1 µg RNA using the RevertAid First Strand cDNA Synthesis Kit (Thermo Scientific). Quantitative RT-PCR was performed with TOPreal™ SYBR Green qPCR Premix (Enzynomics) on a CFX96 Real-Time PCR System (Bio-Rad). *PP2A* served as the internal reference gene. The relative expression levels of *ORE1* and *SAG12* were calculated using the 2⁻^ΔΔCt^ method. The primer sequences are listed in Supplementary Table S1.

### Yeast-two-hybrid (Y2H) assay

The full-length *GI* coding region was cloned into pDEST22 (prey; GAL4 activation domain), and *SOS2*, *WRKY1, 45, 53,* and *75* were inserted into pDEST32 (bait; GAL4 DNA-binding domain) using Gateway cloning. Construct pairs were co-transformed into *Saccharomyces cerevisiae* strain PJ69-4A by PEG-mediated heat shock (42 °C). The transformants were selected on glucose-based synthetic minimal (SD) medium (0.67% yeast nitrogen base, 2% (w/v) glucose, and amino acid dropout solution) lacking leucine and tryptophan (−Leu/−Trp) to select for both plasmids. Interactions were analyzed using −LTH (−Leu/−Trp/−His) media. Colony growth was photographed after 5 d at 30 °C.

### Co-immunoprecipitation (Co-IP) assay

*Nicotiana benthamiana* leaves were co-infiltrated with *Agrobacterium tumefaciens* (GV3101) harboring *35Spro:HA-GI* (*pEarlyGate201*) and/or *35Spro:GFP-WRKY75, 35Spro:GFP-WRKY45* constructs (*pMDC43*). Gateway LR recombination cloning was performed for these plasmids generation using Gateway® LR Clonase™ II (Invitrogen) following the manufacturer's instructions. Leaves were harvested 48 h post-infiltration. Total proteins were extracted in buffer containing 100 mM Tris-HCl (pH 7.5), 150 mM NaCl, 0.5% Nonidet P-40, 1 mM EDTA, 3 mM DTT, and protease inhibitors (1 mM PMSF, 5 µg mL⁻¹ leupeptin, 1 µg mL⁻¹ aprotinin, 1 µg mL⁻¹ pepstatin, 5 µg mL⁻¹ antipain, 2 mM Na₂VO₃, 2 mM NaF, and 50 µM MG132). The extracts were incubated with anti-GFP cross-linked to protein A agarose (Invitrogen) for 2 h at 4 °C. After washing three times, the bound proteins were eluted by boiling in SDS sample buffer. Immunoblotting was conducted with anti-HA (1:2000; 3F10, Roche) and anti-GFP (1:5000; ab6556, Abcam) antibodies. The protein band shifted up by about 15 kDa due to strong binding with the antibody light chain.^24^

### Luciferase reporter assay

The reporter plasmid *SAG12pro:LUC* (containing 1 kb promoter fragment upstream of *SAG12* transcription start site), was cloned into *gZXPomegaLUC +* vector and the effector plasmids *35Spro:HA-GI* and *35Spro:WRKY75-HA* was cloned into *pEarlyGate* vectors. Gateway LR recombination cloning was performed for both reporter and effector plasmid generation using Gateway® LR Clonase™ II (Invitrogen) following the manufacturer's instructions. All of the constructs, including the empty-vector control *35Spro:GUS* (*pCAMBIA1301-GUS*), were transformed in *Agrobacterium tumefaciens* GV3101. Co-infiltrated was performed into 4-week-old *N. benthamiana* leaves using disposable 1 ml syringes. After 48 h, the infiltrated leaves were sprayed with D-luciferin, and bioluminescence images were captured using a NightSHADE LB 985 plant imaging system (Berthold Technologies) with Indigo software (v.2.0.5.0, Berthold Technologies). Statistical data represent mean ± SD of four biological replicates. Significance was determined using one-way ANOVA followed by Tukey's HSD post hoc (*p* < 0.033). Graphs were generated with GraphPad Prism 8.

## Supplementary Material

Supplementary material Supplementary table S1.docx

Spplementary material Spplementary online material.pptx
