# Supplementary material for: GIGANTEA mediates WRKY-dependent transcriptional activation of leaf senescence
Source: Plant Signal Behav. 2026 Mar 2;21(1):2639486. doi: 10.1080/15592324.2026.2639486 (PMC12959225; doi:10.1080/15592324.2026.2639486)
Supplement: Supplementary material — Supplementary table S1.docx [file KPSB_A_2639486_SM4495.docx]

| Primer sequence | | |
| --- | --- | --- |
| ORE1-qRT | Forward | TGTCCACGAGTCCAAAGACG |
|  | Reverse | TGTACCGGACGAATCACGAC |
| SAG12-qRT | Forward | ATCCAAAAGCAACTTCTATTACAGG |
|  | Reverse | CCACTGCCTTCATCAGTGC |
| PP2A-qRT | Forward | GCGGTTGTGGAGAACATGATACG |
|  | Reverse | GAACCAAACACAATTCGTTGCTG |

**Supplementary table S1. Primers used in this study**
