# Supplementary material for: GIGANTEA mediates WRKY-dependent transcriptional activation of leaf senescence
Source: Plant Signal Behav. 2026 Mar 2;21(1):2639486. doi: 10.1080/15592324.2026.2639486 (PMC12959225; doi:10.1080/15592324.2026.2639486)
Supplement: Spplementary material — Spplementary online material.pptx [file KPSB_A_2639486_SM4494.pptx]

## Slide 1
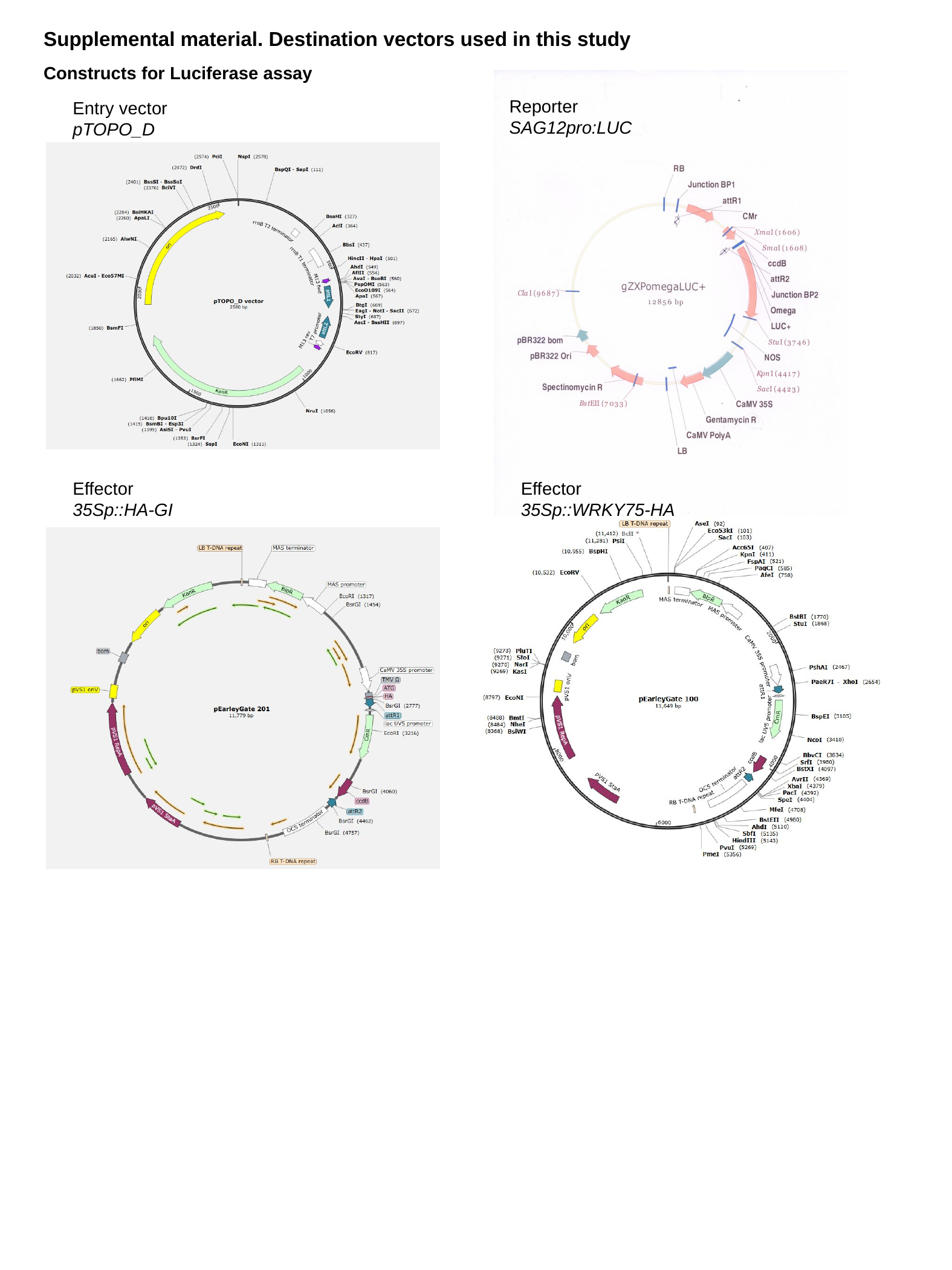

Supplemental material. Destination vectors used in this study
Constructs for Luciferase assay
Reporter SAG12pro:LUC
Entry vector
pTOPO_D
Effector
35Sp::HA-GI
Effector
35Sp::WRKY75-HA

## Slide 2
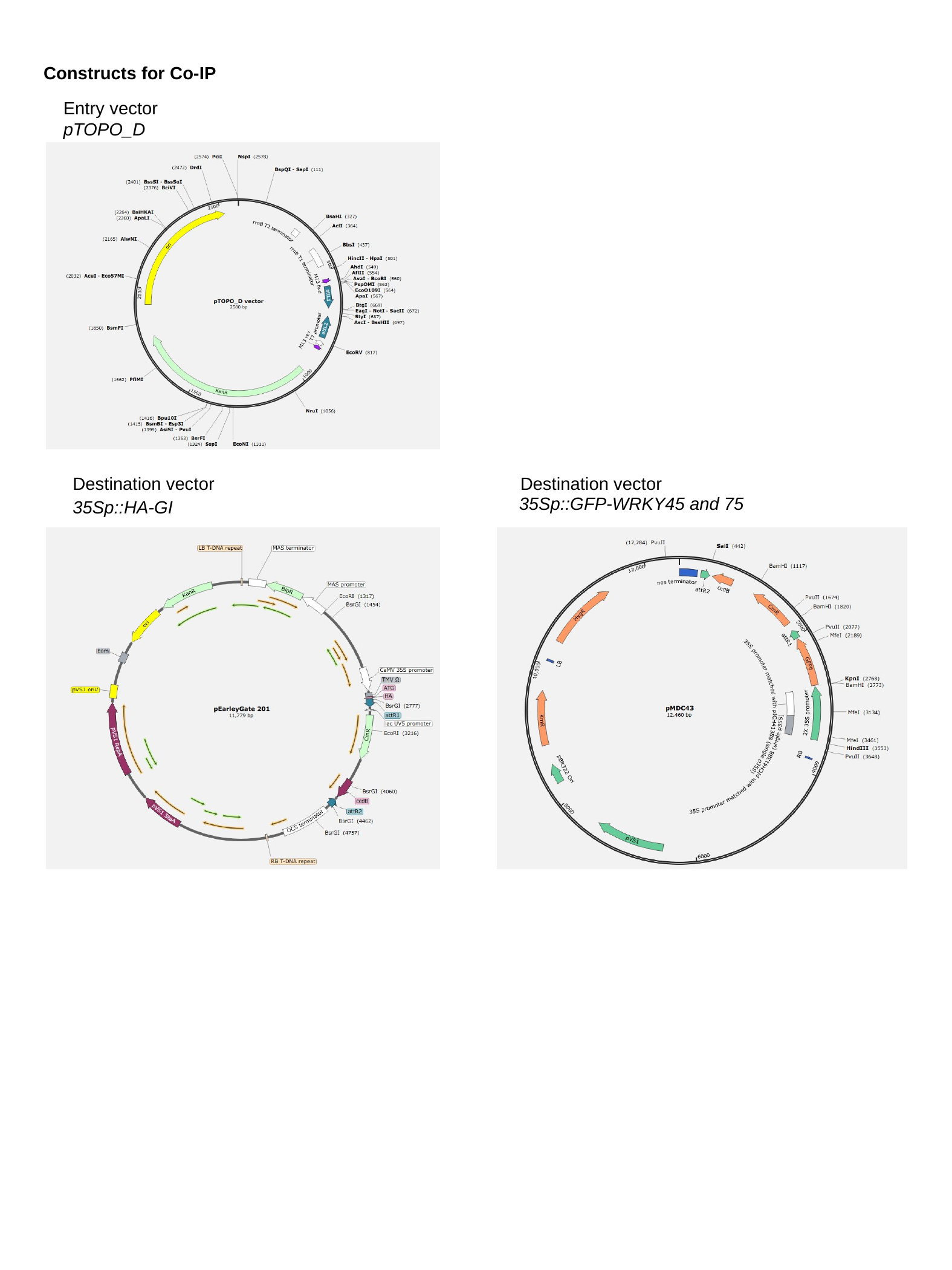

Constructs for Co-IP
Entry vector
pTOPO_D
Destination vector
Destination vector
35Sp::GFP-WRKY45 and 75
35Sp::HA-GI
